# Tubercular Infection Presenting as Sinus Over Ankle Joint after Knee Replacement Surgery

**DOI:** 10.4103/0974-777X.59257

**Published:** 2010

**Authors:** Sanjeev Uppal, Ramneesh Garg

**Affiliations:** *Department of Plastic Surgery, Dayanand Medical College, Ludhiana, Punjab, India*

Sir,

Infection of the joint, following joint replacement surgery, is one of the most dreaded complications. The reason for this is the morbidity and expenditure involved in treating such infections. Common organisms causing infection are coagulase-negative staphylococci, *staph aureus*, mixed flora, *streptococci*, etc. We treated one patient of knee joint replacement, who later developed tuberculous infection. This infection manifested as sinus tract opening at ankle joint. Only one such case has been reported in literature.

**Figure 1:**
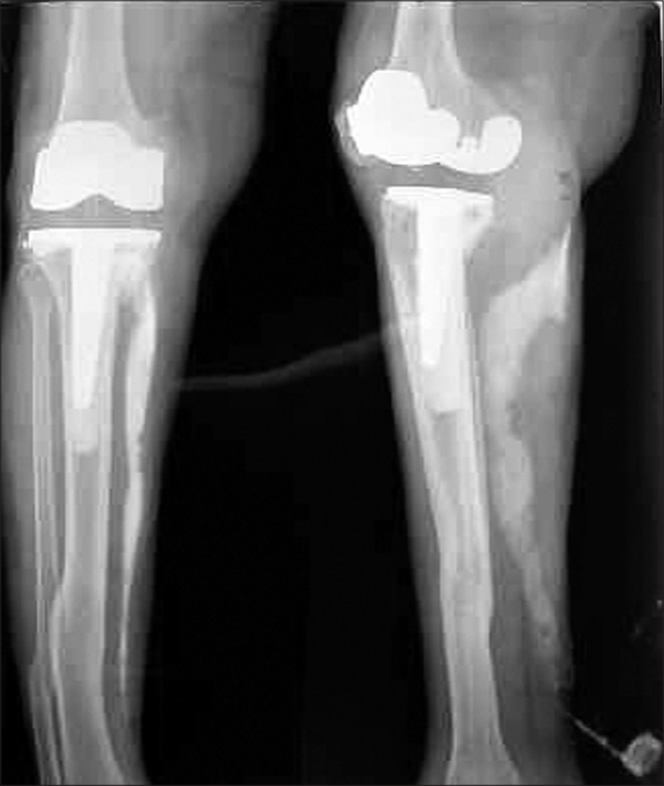
Sinogram showing the sinus tract extending from ankle joint upwards towards knee joint prosthesis

**Figure 2:**
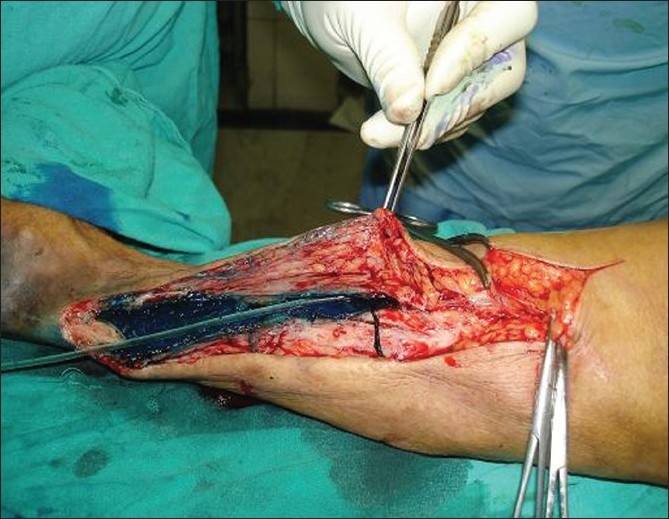
Intraoperative picture with methylene blue dye injected in the sinus

**Figure 3:**
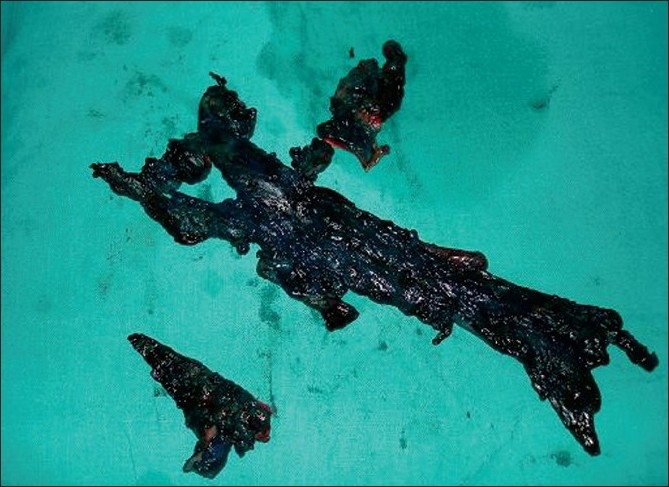
Excised sinus tract

This patient, a 72-year-old woman, presented with complaints of a non-healing wound over inner aspect of right ankle joint for the last 3 years. There was intermittent bloody-to-clear discharge from the wound. The wound used to heal for a short period and then recur spontaneously. There was no history of fever, trauma or purulent discharge. Past history of the patient was very eventful. She had undergone knee replacement surgery in 1987, which had to be repeated because of loosening of prosthesis. It was 1 month after the second surgery that the patient developed swelling in ankle region. The swelling was diagnosed as isolated abscess, which was incised and drained. The patient was put on broad-spectrum antibiotics, but the incision site turned into chronic sinus. Pus cultures were sterile. Blood cultures were not done as it was a localized swelling with no systemic manifestations like fever. Later, biopsy of sinus wall was done, which revealed tuberculosis. Though acid fast bacilli staining was negative, the patient was put on antitubercular treatment for 9 months. The sinus healed temporarily, just to recur after a few months. Local examination showed a sinus opening just above the medial malleolus over the right ankle joint. The surrounding skin was healthy. We observed another 2 × 2 cm-sized firm nontender swelling in the region of medial head of gastrocnemius muscle. Sinogram was done, which revealed sinus tract extending from sinus opening at ankle joint, upwards towards the knee joint [Figure 1]. Excision of sinus tract was planned and patient taken up for surgery. Methylene blue was injected into the sinus tract and dissection made circumferentially around the tract. There was approximately 20 cm long tract starting from medial aspect of knee joint and extending upwards through the gastrocnemius muscle and ending in a closed cavity at ankle joint through which knee prosthesis could be felt [Figure 2]. The whole sinus tract was excised [Figure 3] and the blind cavity at upper end closed. Histopathology of the sinus tract biopsy suggested tuberculosis. The patient was again put on a 9-month course of antitubercular treatment, after which the patient became asymptomatic.

Tubercular infection of the knee joint has been mentioned in literature,[1–3] but infection of a replaced knee joint with *Mycobacterium tuberculosis* is not common.[4] Cases of granulomatous inflammation following joint replacement secondary to foreign body reaction have also been reported in literature.[5] In this case, the possible explanation could be local reactivation of quiescent tuberculosis of the knee joint. The patient had undergone replacement of knee prosthesis due to loosening of previous prosthesis, following fall. Fall might be a coincidental happening, and the actual cause of loosening of prosthesis could be tuberculosis infection. Cause for activation of tuberculosis could be foreign body reaction secondary to metallic prosthesis. There has been only one previous reported case of tuberculosis and foreign body granulomatous reactions involving a total knee prosthesis.[6] In the Indian scenario, where tuberculosis is very common, we strongly recommend pre-surgical diagnostic tests to rule out tuberculosis.
